# Clinical associations and potential novel antigenic targets of autoantibodies directed against rods and rings in chronic hepatitis C infection

**DOI:** 10.1186/1471-230X-13-50

**Published:** 2013-03-19

**Authors:** Laura M Stinton, Robert P Myers, Carla S Coffin, Marvin J Fritzler

**Affiliations:** 1 Liver Unit, Division of Gastroenterology and Hepatology, University of Calgary, Calgary, Alberta, Canada; 2 Department of Medicine, University of Calgary, 3330 Hospital Dr NW, Calgary, Alberta T2N 4N1, Canada

## Abstract

**Background:**

Chronic hepatitis C virus (HCV) infection is frequently associated with extrahepatic autoimmune disorders while interferon (IFN) and ribavirin treatment may exacerbate these conditions. Autoantibodies from HCV patients identify a novel indirect immunofluorescence (IIF) pattern on HEp-2 cells characterized by cytoplasmic rods and rings (RR). Our objectives were to determine the prevalence and clinical associations of RR autoantibodies in HCV patients, and identify related novel autoantibody targets.

**Methods:**

Sera from 315 patients with HCV (301 treatment naive, 14 treated with interferon and/or ribavirin) were analyzed for the presence of RR antibodies by IIF on commercially available HEp-2 cell substrates. Antibodies to inosine monophosphate dehydrogenase 2 (IMPDH2) and cytidine triphosphate synthase 1 (CTPS1) were detected by addressable laser bead assay and other potential targets were identified by immunoscreening a protein microarray. Clinical and demographic data including HCV genotype, mode of infection, prior antiviral therapy, and histological findings were compared between RR antibody positive (RR+) and negative (RR-) patients.

**Results:**

The median age of the HCV cohort was 51 years, 61% were male, and 76% were infected with HCV genotype 1 (G1). Four percent (n=14) had been treated with IFN-based therapy (IFN monotherapy, n=3; IFN/ribavirin, n=11); all had a sustained virologic response. In total, 15 patients (5% of the cohort) were RR+. RR+ and RR- patients had similar demographic and clinical characteristics including age, sex, mode of HCV infection, prevalence of the G1 HCV genotype, and moderate to severe fibrosis. Nevertheless, RR+ patients were significantly more likely than RR- cases to have been treated with IFN-based therapy (33% vs. 3%; adjusted odds ratio 20.5 [95% confidence interval 5.1-83.2]; *P*<0.0005). Only 1/10 RR positive sera had detectable antibodies to IMPHD2 and none had antibodies to CTPS1. Potentially important autoantibody targets identified on protein arrays included Myc-associated zinc finger protein (MAZI) and ankyrin repeat motif.

**Conclusion:**

The majority of HCV patients with RR autoantibodies previously received IFN/ribavirin antiviral therapy. Further studies are necessary to determine the genesis of intracellular RR and elucidate the clinically relevant autoantigens as well as the clinical and prognostic significance of their cognate autoantibodies.

## Background

An estimated 180 million people worldwide are infected with hepatitis C virus (HCV), a known major cause of chronic liver disease [1,2]. HCV infection is also associated with several immunological abnormalities, including the production of both organ specific and non-organ specific autoantibodies [3-5]. Organ specific autoantibodies include those directed against targets in pancreatic islet cells [6], thyroid [7-9], adrenal cortex [6] and gastric parietal cells [10]. Non-organ specific autoantibodies include anti-nuclear antibodies (ANA), anti-smooth muscle antibodies (ASMA), anti-mitochondrial antibodies (AMA), anti-neutrophil cytoplasmic antibodies (ANCA), and anti-liver/kidney microsomal antibodies (LKM) [11-14]. Although their clinical significance remains unclear, ANA have been reported in 4% to 63% of patients with chronic hepatitis C [11,15-18]. Some studies have shown that ANA positivity is associated with stage and rate of fibrosis progression, serum transaminase concentrations and responsiveness to antiviral treatment [19-23]. Other reports have found no differences in these and other clinical parameters [17,24-28]. Interferon (IFN) and ribavirin, cornerstones of the management of HCV infection, have immunomodulatory effects [29,30] such as the production of autoantibodies [31].

In patients with chronic hepatitis C infection, a novel cytoplasmic autoantibody pattern (RR) characterized by rods (~3-10 μm in length) and rings (2–5 μm diameter) has been described on HEp-2 cells [32-34]. Inosine monophosphate dehydrogenase 2 (IMPDH2) and cytidine triphosphate synthase 1 (CTPS1) were identified as potential autoantibody targets localized to the RR structures [32,33]. The objectives of this study were to determine the prevalence and clinical associations of RR autoantibodies in chronic hepatitis C patients, to examine the frequency of antibodies to IMPDH2 and CTPS1 and to identify other potential autoantibody targets by screening high density peptide and protein arrays.

## Methods

### Study cohort

The study cohort included 315 chronic HCV patients followed at the University of Calgary Liver Unit. Three hundred one (301) treatment-naïve patients, all of whom had percutaneous liver biopsies, participated in a study evaluating novel serum biomarkers of liver fibrosis. Sera from these patients were collected prior to liver biopsy and stored at -80°C. The remaining 14 patients were involved in a study evaluating the prevalence of occult HCV and hepatitis B infection. All patients had achieved a sustained virologic response (SVR) to anti-HCV therapy as defined by undetectable HCV RNA 6 months following interferon and/or ribavirin treatment [35]. The sera from these patients were collected after therapy. Clinical and demographic data including age, gender, HCV genotype, mode of HCV acquisition, prior antiviral therapy, biochemical data, and histological findings were obtained by a retrospective review of medical records. Sera from 100 primary biliary cirrhosis (PBC) [36] and 27 systemic lupus erythematosus (SLE) patients were used as controls. The study protocol was conducted in accordance with the Conjoint Health Ethics Review Board at the University of Calgary.

### Indirect Immunofluorescence (IIF)

Serum samples were analyzed for autoantibodies at the Mitogen Advanced Diagnostics Laboratory, University of Calgary (http://www.mitogen.ca). RR autoantibodies [37] were detected by IIF on HEp-2 cells (human laryngeal carcinoma cell line: INOVA Diagnostics, Inc.; San Diego, CA) using the manufacturer’s protocol, which included serum diluted to 1/160 and a heavy chain-specific, fluorescein-conjugated goat anti-human immunoglobulin IgG as the secondary antibody. For comparison purposes, sera were also tested on a HEp-2 substrate from another manufacturer (ImmunoConcepts Inc., Sacramento, CA) using their recommended protocol. In addition to the RR pattern, other IIF patterns (e.g. ANA, AMA) were also recorded. All slides were viewed by an experienced technologist on a Zeiss Axioshop 2 Plus microscope (Carl Zeiss Inc., Thornwood, NY) fitted with appropriate filters. Images were recorded using a Zeiss Axiocam HRc digital camera and Zeiss Axiovision (version 3.1) software. Digital images were processed using Photoshop Version 12.1 (64 bit).

### Tissue culture cells

HEp-2 (Dr. Edward K.L Chan, University of Florida) and HeLa (human cervical cancer; American Type Culture Collection (ATCC: Manassas, VA) cells were cultured in DMEM containing 10% Fetal Bovine Serum (FBS) and 1% penicillin-streptomycin; CHO (Chinese hamster ovary; ATTC) cells were grown in RPMI 1640 with 10% FBS and 1% penicillin-streptomycin media. All cells were cultured on glass slides or cover slips in a 37°C incubator under 5% CO_2_. Before cells reached confluence, they were fixed and permeabilized with a variety of fixatives including 3.5% paraformaldehyde at room temperature for 30 minutes followed by a rinse in phosphate buffered saline (PBS) and then permeabalized with 0.5% Triton X100. Other organic fixatives included ice-cold acetone, methanol and a methanol-acetone mixture (3:1 volume:volume) for 15 minutes followed by air drying the slides to at room temperature. The nuclei of CHO cells were counterstained with 4′,6-diamidino-2-phenylindole (DAPI). In an attempt to determine the tissue culture conditions that were responsible for the RR pattern, HEp-2 and HeLa cells were treated with ribavirin (Sigma-Aldrich; R9644) at a concentration of 1 μM for 1–3 hours as previously reported [33]. These cell preparations were then processed for IIF using the reagents and protocols as described above for the HEp-2 slides obtained from Dr. Edward K.L. Chan.

### IMPDH2 and CTSP1 immunoassay

Purified, full length human IMPDH2 (Abnova; Taipei City, Taiwan) and full length recombinant CTSP1 (NOVUS Biologicals, Littleton, CO, USA: Catalogue # AAH09408) were covalently coupled to addressable laser beads (Luminex Corporation, Austin, TX) as previously described [36]. These coupled beads were then used to develop an immunoassay with respective monoclonal antibodies (Abcam; Cambridge, MA) as the positive markers and normal human sera as negative controls using a Luminex 200 fluorometer (Luminex Corp.) according to previously published protocols [38].

### Protein arrays

Human peptide arrays containing ~30,000 proteins spotted in duplicate on solid phase membranes (imaGenes, Berlin, Germany) were screened by an immunoblotting protocol provided by the manufacturer using two RR index sera and normal control sera to detect novel biomarkers of interest in RR sera. Briefly, the membrane was rinsed in 70% ethanol, rinsed in distilled water and then hydrated in Tris buffered saline with Tween20 (TBST: 10 mM Tris/Cl pH7.6, 150 mM NaCl, 0.1% Tween20) and then immersed in the provided blocking solution. After overnight incubation at 4°C, the membrane was washed three times at 15 min intervals with TBST and then immersed in the blocking solution. The RR sera and control sera with unrelated autoantibody activity were diluted 1/100 in the blocking solution and separately applied to the membrane, incubated for 2 hr at room temperature, washed in three changes of TBST and then in TBS to remove the detergent. Horse radish peroxidase (HRP)-conjugated anti-human or anti-mouse antibodies (Jackson ImmunoResearch Lab, West Grove, PA), was diluted 1/10,000 according to the manufacturer’s protocol and the bound antibodies visualized with enhanced chemiluminescence western blotting reagents (Amersham Biosciences, Baie d’urfe, Quebec). The reactive peptides were identified by referring to an array identification grid provided by the manufacturer and were distinguished from non-specific and unrelated peptides by comparison to the control sera.

### Statistical analyses

Demographic, clinical and histologic characteristics of the study cohort were described using the median [interquartile range (IQR)] and proportions. Comparisons between groups (e.g. RR antibody positive versus negative) employed Fisher’s exact tests for categorical variables and Mann–Whitney tests for continuous. Univariate and multivariate logistic regression analyses examined predictors of RR antibody positivity. Due to the limited number of RR antibody positive cases, the multivariate model included only age, gender, HCV genotype and prior antiviral treatment. All statistical analyses were performed using Stata version 10.1 software (Stata Corp., College Station, TX, USA). Two-sided *P*-values less than 0.05 were considered statistically significant.

## Results

### Study population

The characteristics of the study population are outlined in Table 1. The median age of the 315 HCV-infected patients was 51 years (IQR 45–54); 61% were male, 76% were infected with HCV genotype 1, and 45% had acquired HCV via injection drug use. In total, 14 patients (4.4%) had previously been treated with IFN-based therapy, 3 with IFN monotherapy and 11 with IFN and ribavirin combination therapy. All treated patients achieved an SVR. Liver biopsies revealed moderate to severe necroinflammatory activity (A2-3) and fibrosis (F2-4) in 72% and 66% of patients, respectively.

**Table 1:** Demographic, clinical, and autoantibody features of the study population (n=315)

**Characteristic**	Frequency (N)*
**Demographics**	
Median age, *yrs*	51 (45–54)
Male gender	61% (193)
**HCV genotype**	
1	76% (239)
2 and 3	18% (58)
Other/unknown	6% (18)
**Mode of HCV infection**	
Injection drug use	45% (141)
Blood transfusion	19% (59)
**Prior HCV treatment**	4% (14)
Interferon monotherapy	21% (3/14)
Interferon and ribavirin	79% (11/14)
**Biochemical data **^†^	
ALT, IU/L	64 (42–110)
Platelets, x10^9^/L	200 (163–243)
**Histologic characteristics**	
A2-3 necroinflammation	72% (226)
F2-4 fibrosis	66% (208)
Cirrhosis (F4)	9% (27)
**Autoantibodies**	
RR positive	5% (15)
ANA positive	89% (281)
Anti-mitochondrial antibody positive	2% (5)

* All data are median (IQR) or proportions (%, n).

^† ^ALT and platelets missing in 10 and 5 patients, respectively.

Abbreviations – ANA, antinuclear antibodies; AMA, anti-mitochondrial antibodies; RR, Rods and Rings.

### Rods and rings (RR) antibodies

**Figure 1:**
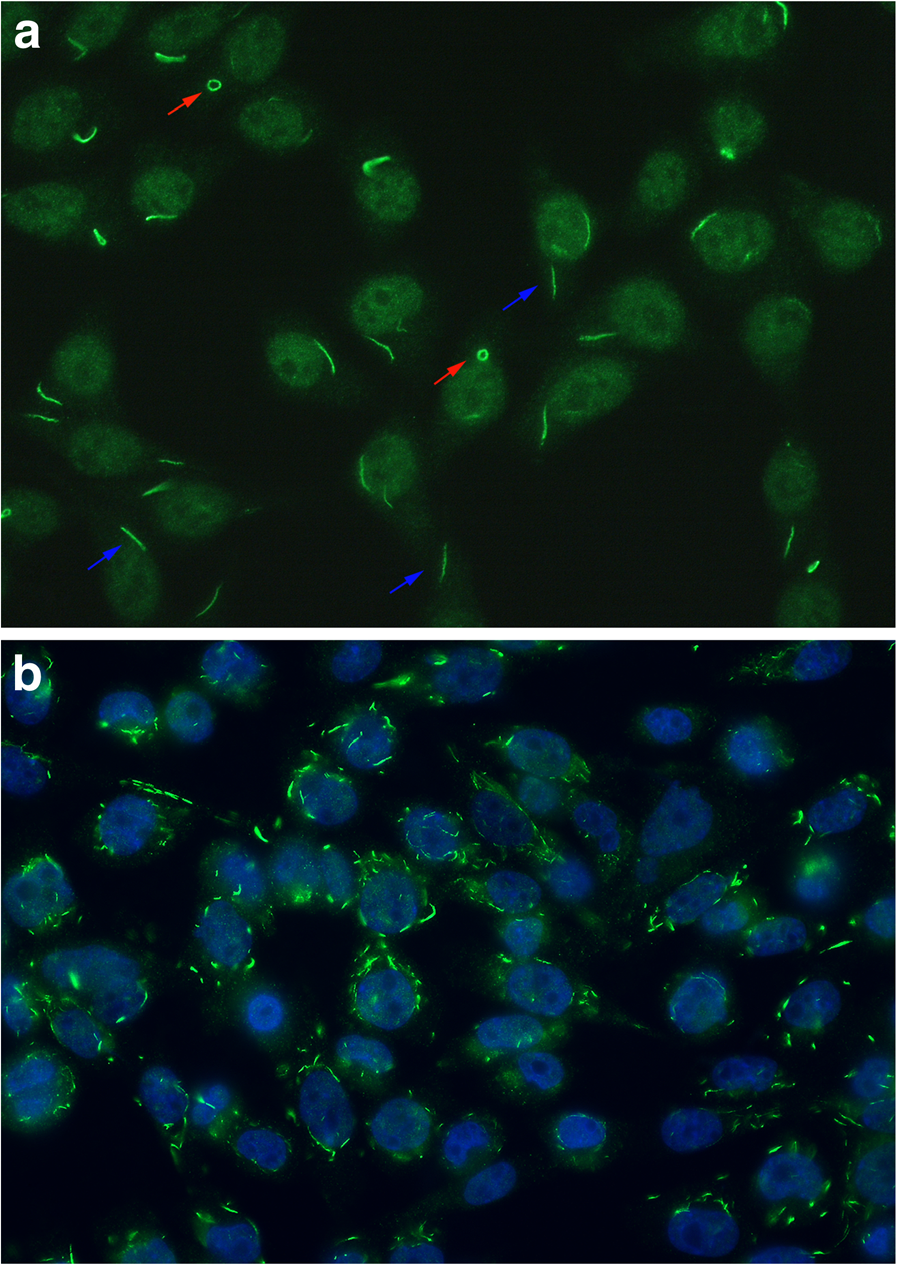
**a) Indirect Immunofluorescence (IIF) on HEp-2 cells of a prototype human serum with autoantibodies directed to rods (blue arrows, ~3-10 μm in length) and rings (red arrows, 2–5 μm diameter) (RR). b**) Chinese hamster ovary cells (CHO) constitutively express multiple cytoplasmic structures/cell primarily represented as rods of various lengths. Fixation 3.5% paraformaldehyde; nuclei counterstained blue with DAPI.

IIF on HEp-2 cell substrates from INOVA Diagnostics revealed the presence of the RR staining pattern in 15/315 (4.8%) sera (Figure 1a). However, when the sera were retested on ImmunoConcepts HEp-2000 substrate the RR staining pattern was not observed (not shown). None of the various cell fixatives tested (2% buffered paraformaldehyde/Triton X permeabilization, acetone, methanol, acetone:methanol mixtures) produced the RR pattern in commercially available multi-passaged HEp-2 or HeLa cells. Considering that the staining might be related to a specific subclone of cells, HEp-2 cells known to produce the RR pattern were obtained from Dr. Edward K.L. Chan (University of Florida) and cultured under identical conditions. In these cells, RR staining identical to that illustrated in Figure 1 was seen with a variety of fixation protocols but was best preserved by fixation in 2% buffered paraformaldehyde. However, with 2 to 3 successive cell passages the RR pattern disappeared. Of the other cell lines tested (i.e. CHO and HeLa) only CHO cells were found to constitutively produce RR represented primarily as numerous cytoplasmic rod- or cane-like like structures of various lengths (Figure 1b). Of note, treatment of HeLa and HEp-2 cells with 1 μM ribavirin for 3 hours induced the formation of RR in HeLa and HEp-2 cell lines.

### Other autoantibodies, anti-IMPDH2 and candidate RR antibody targets

A total of 281 (89%) HCV sera tested positive for autoantibodies by IIF on HEp-2 substrates; a nuclear staining pattern was the most common (76%, 238/315). Only five patients (2%) had an AMA IIF staining pattern. When 10 RR IIF positive sera were tested by ALBIA, only 1 had antibodies to IMPDH2 and none reacted with CTSP1. Neither IMPDH2 nor CTPS1 antibodies were detected in the PBC, SLE, or normal control sera.

When the protein microarray was screened with the RR sera and compared to results from normal controls and unrelated autoantibodies, a number of targets of interest were identified (Table 2). The strongest and most consistent signals were derived from four separate clones all related to Myc-associated zinc finger protein (MAZI: SWISSPROT Accession # P56270). In addition, reactivity to voltage-dependent anion channel 1 (VDAC1), ankyrin repeat and sterile alpha motif domain containing 6 (ANKS6), ARP1 actin-related protein 1 homolog A and three unnamed peptides of unknown function.

**Table 2:** Potential RR autoantibody targets indentified on a protein/peptide microarray

**Clone ID**	**Description**	**Structure/function**	Reactivity*
MPMGp800M18568 MPMGp800E06542 MPMGp800H07541 MPMGp800P08580	Homo sapiens MAZI; Alternate name: Purine- binding transcription factor (ZF87) (ZIF87). Source: SWISSPROT; Acc:P56270	C2H2-type zinc fingers; transcription initiation and termination; purine metabolism [39,40]	4+
MPMGp800O22578	Homo sapiens VDAC1	Outer mitochondrial membrane; role in apoptosis and cancer; interacts with amyloid and tau role in Alzheimer’s [41-43]	3+
MPMGp800A24548	Homo sapiens ARM and sterile alpha motif domain containing 6 (ANKS6)	ARM participate in protein folding and found in ~6% of eukaryotic proteins [44,45]	2+
MPMGp800L08582	Homo sapiens ARP1 – homolog A (ACTR1A)	Major subunit of dynactin; molecular motor component; binds ATP and NuMA [46,47]	3+
MPMGp800K12532 MPMGp800L22599 MPMGp800E04593	Unknown proteins	Unknown	2 – 3+

*Reactivity = the intensity of immunoreactivity with proteins on the microarray which were graded from 0 (no reactivity) to 4 (high intensity).

Abbreviations: ARM, ankyrin repeat motif; ARP, actin-related protein; ATP, adenosine triphospate; MAZI, Myc-associated zinc finger protein; NuMA, nuclear mitotic apparatus (NuMA) protein; VDAC1, voltage-dependent anion channel 1.

### Clinical associations of rods and rings (RR) antibody positivity

As shown in the Table 3, HCV-infected patients with and without RR antibodies had similar demographic and clinical characteristics including age, gender, infection with HCV genotype 1, mode of HCV acquisition, serum ALT concentration, platelet count, and severity of necroinflammation and fibrosis. Positivity for other autoantibodies including ANA and AMA IIF staining patterns did not differ between RR positive and negative cases. However, RR antibody-positive patients were significantly more likely to have previously received IFN-based treatment (33% vs. 3%; OR 16.2 [95% CI 4.6-57.1]; *P*<0.0005). Whereas 5 of 11 (45%) patients treated with IFN and ribavirin combination were RR antibody positive, none of the 3 patients who received IFN monotherapy had RR antibodies (*P*=0.26). In a multivariate logistic regression analysis including age, sex, and HCV genotype, prior IFN treatment was the only independent predictor of RR antibody positivity (OR 20.5 [95% CI 5.1-83.2]; *P*<0.0005). Since all patients treated with IFN achieved an SVR, the effect of treatment success on the presence of RR autoantibodies could not be determined.

**Table 3:** Predictors of rods and rings antibody (RR) positivity

**Characteristic**	**RR negative (n=300)**	**RR positive (n=15)**	***P*-value**
**Demographics**			
Median age, *yrs*	51 (44–54)	51 (46–61)	0.38
Male gender	61% (182)	73% (11)	0.42
**HCV genotype 1**	76% (228)	73% (11)	0.76
**Prior injection drug use**	45% (134)	47% (7)	1.00
**Prior HCV treatment**	**3% (9)**	**33% (5)**	**<0.0005**
**Biochemical data **^†^			
ALT, IU/L	65 (42–110)	52 (24–80)	0.16
Platelets, x10^9^/L	202 (167–243)	182 (134–245)	0.38
**Histologic characteristics**			
A2-3 necroinflammation	73% (218)	53% (8)	0.14
F2-4 fibrosis	66% (198)	67% (10)	1.00
**Autoantibodies**			
ANA positive	89% (266)	100% (15)	0.39
AMA positive	2% (5)	0% (0)	1.00

Abbreviations: ANA, antinuclear antibodies; AMA, anti-mitochondrial antibodies; RR, Rods and Rings. † ALT and platelets missing in 10 and 5 patients, respectively.

## Discussion

HCV infection is associated with a wide spectrum of immune reactions, some of which are reflected by the presence of organ and non-organ specific autoantibodies. Possible mechanisms for the production of autoantibodies include molecular mimicry, an interaction of the HCV with B lymphocytes promoting B cell proliferation and activation, or direct infection of immunocytes by HCV [48-52]. In addition, patients receiving interferon and/or ribavirin therapy may have accelerated pre-existing autoimmune diseases, or *de novo* occurrence of autoimmune disorders or autoantibody production [26,31,53-58].

A novel autoantibody staining pattern has recently been reported in patients with HCV infection characterized by rods (~3-10 μm in length) and rings (2–5 μm diameter) localized to the cytoplasm of certain cell lines and expresed throughout the cell cycle [32-34]. Other studies have determined that this IIF pattern is associated with antibodies directed against IMPDH2 or CTPS1 [32,33,59]. In our study we confirmed that IMPDH2 reacts with a minority of HCV sera, a finding in keeping with reports by others [33,59]. Although CTSP1 was localized to RR [33], it does not appear to be a primary target of human autoantibodies as none of our sera in this study or human sera in a previous study [33] reacted with the purified CTSP1 protein.

While the frequency of the reactivity to IMPDH2 in the present study is less than previously reported [32,33,59], it is clear from studies to date that other autoantibody targets remain to be identified. To address this possibility, we probed a commercially available protein and peptide microarray and identified a number of unique potential autoantibody targets (Table 2), where the Myc-associated zinc finger protein (MAZI) is of particular interest [39]. There is evidence that MAZI, which contains six C2H2-type zinc fingers, functions as a transcription factor with dual roles in transcription initiation and termination [40]. While the cellular localization has not been definitively determined, it is presumed to be primarily localized to the nucleus, although in brains of Alzheimer disease patients it is localized to plaque-like structures in the cytoplasm [60]. Of note, MAZI is expressed in kidney, liver and brain and it is a purine binding transcription factor. The latter feature is of particular interest because of its potential relation to inosine metabolism and IMPDH2 previously identified RR autoantibody targets [32,33,59].

The actin-related protein Arp1 (or centractin) is the major subunit of dynactin, a key component of the cytoplasmic dynein molecular motor [46]. Under certain conditions Arp1 has high homology to conventional actin, which has been shown to polymerize [46]. Arp1 is also predicted to bind ATP and another autoantibody target, the nuclear mitotic apparatus protein (NuMA) [61]. Likewise, the ankyrin repeat motif (ARM) identified as part of the sterile alpha motif domain containing 6 (ANKS6) protein is of interest. ARMs are typically comprised of 33 residues and are structurally represented as two alpha helices separated by loops [44,45]. ARM is also one of the most common protein–protein interactions that mediate protein-protein interactions and several unique aspects of protein folding [44,45]. Ankyrin repeats appear in virtually all organisms but are most abundant in eukaryotic cells where they are found in 6% of proteins of diverse function such as transcriptional initiators, cell cycle regulators, cytoskeleton, ion transporters, and signal transducers. The voltage-dependent anion channel 1 (VDAC1) localized to the outer mitochondrial membrane has been shown to control metabolic interactions between mitochondria and the rest of the cell [41]. VDAC1 has been implicated in the control of apoptosis, including via its interaction with the pro- and anti-apoptotic proteins [41,42] and due to an abnormal interaction with amyloid beta and phosphorylated tau, is implicated in mitochondrial dysfunction in Alzheimer’s disease [43]. VDAC1 also contributes to the metabolic phenotype of cancer cells as reflected by its over-expression in many cancer types [41]. Whereas these candidate target autoantigens have common structural and functional properties (i.e. purine metabolism and protein folding, aggregation and polymerization), additional studies are needed to establish immunoassays and determine the prevalence of antibodies to these novel targets identified in our study of index RR sera.

In our study of various cell lines, commercially available HEp-2 substrates from INOVA Diagnostics and CHO cells maintained in our own tissue culture facility constitutively expressed RR. Others have also reported that the HEp-2 substrate provided by INOVA seems to be unique in demonstrating “out of the box” RR staining [34]. Of interest, a HEp-2 cell line obtained from Dr. Edward K.L. Chan, one of the first to report the RR pattern, produced the RR pattern but after 2 to 3 passages of these cells, RR became undetectable. This suggests that under certain growth or tissue culture conditions, RR expression can either be diminished or facilitated. However, when a variety of tissue culture protocols including various antibiotics, media and heat shock were used, we were unable to demonstrate RR formation. Since tissue culture and fixation protocols are considered a trade secret by manufacturers of HEp-2 diagnostic substrates, the reasons for this finding are not fully understood. As reported by others [32,34], we confirmed that the RR pattern is restored after cells are treated with ribavirin. Of interest, ribavirin is likely not an obligatory reagent in all cells since we are the first to report that CHO cells, constitutively and without adding exogenous ribavirin, express similar immunoreactive RR structures.

In the present study, the 5% frequency of RR autoantibodies in our HCV cohort is lower than the 20-35% prevalence previously described in HCV sera [32,34]. The finding of antibodies to RR appears to be relatively specific because we did not identify any RR autoantibodies in PBC or SLE sera. Retrospective chart review did not find an association between RR autoantibodies with clinical characteristics including age, gender, mode of HCV infection, prevalence of HCV genotype 1, serum ALT concentration, platelet count, severity of necroinflammation and fibrosis or the presence of either ANA or AMA. However, both uni- and multi-variate analysis showed that prior HCV IFN and ribavirin treatment was the only independent predictor of RR antibody positivity. Since none of the patients treated with IFN monotherapy had RR autoantibodies, RR autoantibodies seems to only be present in HCV patients treated with combination IFN and ribavirin therapy. This is supported by studies reported here and by others showing that RR are induced after treatment of cell lines with ribavirin, but not IFN [33]. These results also confirm previous reports that RR autoantibodies were significantly associated with prior IFN/ribavirin treatment [34]. Other studies have shown that anti-RR antibodies were not present at disease baseline, but appeared during IFN/ribavirin therapy and were more often detected in non-responder/relapsers than in responder patients [59]. Nevertheless, it is clear the antibodies to RR are seen in patient sera that have no obvious HCV infections or treatment with ribavirin or IFN [32], suggesting that other mediators are likely involved in induction of the B cell anti-RR response. As all of our patients treated for HCV achieved an SVR, we were unable to assess this in our study but is amenable to more effective analysis in multi-center studies of larger cohorts because it may be a biomarker for poor response to therapy. In addition, the effect of ‘triple therapy’ on the induction of RR autoantibodies, has yet to be investigated.

Limitations to our study include the small sample size of HCV treated patients and the majority of our cohort were not treated with IFN/ribavirin at the time of sera collection (convenience sample as described above). However, this may be an advantage in showing the importance of IFN/ribavirin as a triggering or modulating factor in the induction of these novel autoantibodies as it does support previous studies, that RR are primarily seen in IFN/ribavirin treated patients thus explaining our relatively low frequency of RR as compared to previous reports. The fact that all treated patients achieved an SVR, limited our ability to fully identify the relationship of RR autoantibodies to treatment outcomes. In addition, this is a retrospective study without longitudinal sera samples (i.e. pre, during, and post therapy).

## Conclusion

In conclusion, a novel cytoplasmic autoantibody staining pattern, rods and rings (RR) has recently been reported in patients with HCV infection [32,34]. We identified this pattern in 5% of our HCV cohort and found that prior IFN and ribavirin treatment was significantly associated with RR autoantibody positivity. Only a minority of our RR sera reacted with IMPDH2 and none reacted with CSP1, suggesting more effort is required in identifying the related target autoantigens. Further investigations are warranted to further determine the clinical, pathogenic, and prognostic significance of autoantibodies directed against RR.

## Abbreviations

ANA: Antinuclear antibodies; AMA: Anti-mitochondrial antibodies; CHO: Chinese hamster ovary cells; HCV: Hepatitis C virus; IFN: Interferon; PBC: Primary biliary cirrhosis; RR: Rods and rings; SLE: Systemic lupus erythematosus; SVR: Sustained virologic response.

## Competing interests

M.J. Fritzler is a consultant to Glaxo Smith Kline Canada, Pfizer, ImmunoConcepts, BioRad, Euroimmun GmbH, Dr. Fooke Laboratorien GmbH, and INOVA Diagnostics, Incorporated. The other authors have no disclosures.

## Authors’ contributions

LMS conceived of the study, performed the clinical analysis and chart reviews, compiled the database and participated in drafting, compiling and editing the manuscript; RPM and CSC provided clinical material and sera, assisted with the study design and edited the manuscript; MJF conceived of the study, conducted the serological analysis, conducted immunofluorescence and participated in drafting, compiling and editing the manuscript. All authors read and approved the final manuscript.

## Pre-publication history

The pre-publication history for this paper can be accessed here:

http://www.biomedcentral.com/1471-230X/13/50/prepub
